# Unmasking Sarcoidosis: Fever and Arthralgia Revealing an Underlying Granulomatous Disease

**DOI:** 10.7759/cureus.99535

**Published:** 2025-12-18

**Authors:** Ana Maria Carvalho, Pedro Sá Almeida, Rita G Magalhães, Tiago Silveira-Rosa, João Enes Silva, Sofia Perdigão

**Affiliations:** 1 Internal Medicine, Unidade Local de Saúde de Trás-os-Montes e Alto Douro, Chaves, PRT

**Keywords:** arthralgia, extrapulmonary sarcoidosis, fever of unknown origin, hepatic sarcoidosis, sarcoidosis

## Abstract

Sarcoidosis is a multisystem granulomatous inflammatory disease of unknown etiology, with a highly variable clinical presentation. Diagnosis can be particularly challenging, as the condition may manifest through a wide range of nonspecific symptoms, involve multiple organs, and lack pathognomonic findings.

We describe the case of a previously healthy 65-year-old man who presented to the emergency department with fever and inflammatory large-joint arthralgias, without other associated symptoms. Physical examination was unremarkable, with no palpable lymphadenopathy, rash, or inflammatory signs in any joints examined. Initial laboratory evaluation showed a cholestatic pattern of liver enzymes, increased C-reactive protein, and elevated angiotensin-converting enzyme. Chest computed tomography (CT) revealed mediastinal-hilar lymphadenopathy. During hospitalization, the patient maintained a persistent fever and arthralgias, prompting further evaluation with 18F-fluorodeoxyglucose positron emission tomography-computed tomography (18F-FDG PET-CT), which demonstrated symmetric FDG uptake in thoracic lymph nodes and peripheral joints. Given the unexplained cholestatic pattern, a liver biopsy and lymph node biopsy were performed; there were no findings compatible with granulomas or malignancy. After excluding infectious, autoimmune, and neoplastic causes, the hypothesis of sarcoidosis persisted even with no definitive histological support, and the diagnosis was made through bronchoalveolar lavage (BAL), which revealed lymphocytic inflammation with a CD4/CD8 ratio of 7.3, highly compatible with sarcoidosis. Corticosteroid therapy was initiated with an excellent clinical response.

This case highlights the importance of a systematic and comprehensive diagnostic approach when evaluating prolonged fever and inflammatory arthralgias. It underscores the need to consider sarcoidosis, even as a diagnosis of exclusion, particularly in atypical presentations with predominant joint involvement.

## Introduction

Sarcoidosis is a multisystem granulomatous disorder of unclear etiology, characterized histologically by non-caseating granulomas and capable of affecting virtually any organ system. These granulomas arise from an exaggerated cell-mediated immune response, although the precise trigger remains unknown. Although pulmonary involvement is the most common presentation, extrapulmonary disease occurs in 30-50% of patients and may include hepatic, cutaneous, ocular, cardiac, and musculoskeletal manifestations [1,2]. This clinical heterogeneity often complicates diagnosis, particularly when respiratory symptoms are absent or subtle, and diagnostic confirmation may be further challenged by nondiagnostic biopsy specimens, as granulomas can be limited or entirely absent in early or predominantly extrapulmonary disease [3,4].

The diagnosis of sarcoidosis typically relies on three pillars: compatible clinical and radiologic findings, evidence of non-caseating granulomas when obtainable, and exclusion of alternative causes such as infections or autoimmune disease [1].

Hepatic sarcoidosis is frequently under-recognized because it is often subclinical; however, patients may present with cholestatic liver enzyme abnormalities, hepatomegaly, or, less commonly, portal hypertension and advanced fibrosis [5]. The variability of histologic findings - ranging from well-formed granulomas to patchy or nondiagnostic samples - can further hinder timely diagnosis, as liver biopsy may be normal despite significant clinical involvement [5,6].

Rheumatologic manifestations are also common, with arthralgias, acute arthritis, and periarticular inflammation occurring in up to one-third of patients and sometimes preceding pulmonary involvement, often prompting evaluation for infectious or autoimmune conditions [7]. Additional diagnostic tools such as bronchoalveolar lavage (BAL) may provide supportive information - particularly an elevated CD4/CD8 ratio - although this finding is not specific and must be interpreted in a clinical context [8].

Advanced imaging, particularly 18F-fluorodeoxyglucose positron emission tomography-computed tomography (18F-FDG PET-CT), plays an increasingly important role in identifying active granulomatous inflammation, mapping disease extent, and excluding alternative diagnoses such as malignancy. Symmetric hypermetabolic activity in thoracic lymph nodes and involved organs is a typical pattern in sarcoidosis [1,9].

The present case highlights these diagnostic complexities, as the patient initially presented with fever, inflammatory arthralgias, and cholestatic liver enzyme abnormalities without an obvious focus. A broad multidisciplinary evaluation was required to establish the diagnosis of sarcoidosis, emphasizing the importance of maintaining a wide differential diagnosis when encountering atypical systemic inflammatory presentations.

## Case presentation

We present the case of a 65-year-old man with a history of dyslipidemia and a tubular gastric adenocarcinoma under endoscopic surveillance (pT1aR0). He was a former smoker and reported no significant alcohol consumption.

He presented to the emergency department with vespertine fever (38°C), measured by auricular thermometry, for one week, associated with fatigue. He reported recurrent episodes of inflammatory arthralgias involving the shoulders and hips for approximately six months, often accompanied by mild fever, with temporary relief after ibuprofen. He denied dyspnea, chest pain, weight loss, night sweats, skin lesions, headache, visual changes, gastrointestinal symptoms, recent travel, ingestion of unpasteurized foods, contact with animals, or recent medications/vaccinations.

On physical examination, he was hemodynamically stable and in good general condition. There was no rash, jaundice, Janeway lesions, splinter hemorrhages, erythema nodosum, palpable lymphadenopathy, abdominal tenderness, hepatosplenomegaly, or inflammatory joint signs. Cardiopulmonary auscultation was normal, with no murmurs.

Laboratory studies revealed a cholestatic pattern of liver enzyme abnormalities and elevated inflammatory markers. Complete blood count, renal function, and electrolytes were normal. Angiotensin-converting enzyme (ACE) was elevated, as was ferritin (Table 1). These abnormalities - particularly the cholestatic profile, marked inflammatory response, and elevated ACE - were clinically meaningful, as they raised early suspicion for a systemic inflammatory or granulomatous disease rather than an isolated hepatobiliary or infectious process.

**Table 1:** Laboratory evaluation. ACE: angiotensin-converting enzyme; ALP: alkaline phosphatase; ALT: alanine transaminase; AST: aspartate transaminase; CRP: C-reactive protein; ESR: erythrocyte sedimentation rate; GGT: gamma-glutamyl transferase; Hb: hemoglobin

Laboratory parameter	Admission	Follow-up	Clinical discharge	Reference values
Hb	12.5 g/dL	11.1 g/dL	14.00 g/dL	13.00-18.00 g/dL
AST	111 U/L	120 U/L	25 U/L	<40 U/L
ALT	167 U/L	190 U/L	33 U/L	<41 U/L
GGT	757 U/L	800 U/L	49 U/L	10-49 U/L
ALP	376 U/L	400 U/L	70 U/L	40-130 U/L
CRP	10.1 mg/dL	15.1 mg/dL	<0.5 mg/dL	<0.5 mg/dL
ESR	73 mm/h	-	7 mm/h	0-20 mm/h
ACE	70 U/L	-	35 U/L	20-70 U/L
Ferritin	1000 ng/mL	1400 ng/mL	400.0 ng/mL	30.0-400.0 ng/mL

The autoimmune panel (antinuclear antibodies (ANA), anti-cyclic citrullinated peptide (anti-CCP), rheumatoid factor, anti-mitochondrial antibodies, anti-liver cytosol type 1 (anti-LC1), anti-liver kidney microsomal type 1 (anti-LKM1), anti-smooth muscle antibodies, liver-specific autoantibodies, anti-SSA/SSB, and antineutrophil cytoplasmic antibodies (ANCA)) was negative, as were serologies for HIV-1/2 and hepatitis C and E. Hepatitis A and B, cytomegalovirus (CMV), Epstein-Barr virus (EBV), and toxoplasmosis were compatible with past infection. Complement levels were normal. Thyroid function tests, serum IgG, IgM, IgA, and serum protein electrophoresis were all within normal ranges (Table 2). These studies were performed to exclude autoimmune, infectious, and other systemic conditions that may mimic sarcoidosis in early or extrapulmonary presentations.

**Table 2:** Comprehensive laboratory workup performed to investigate infectious, autoimmune, and inflammatory etiologies during the diagnostic evaluation. AMAs: anti-mitochondrial antibodies; ANAs: antinuclear antibodies; ANCAs: anti-neutrophil cytoplasmic antibodies; C3 and C4: complement C3 and C4; CMV: Cytomegalovirus; EBV: Epstein-Barr virus; ENAs: extractable nuclear antigen antibodies (the ENA screening test detects antibodies against dsDNA, SS-A (Ro), SS-B (La), Sm, RNP, Scl-70, centromere, Jo-1, fibrillarin, RNA polymerase III, PM-Scl, PCNA, Mi-2, and ribosomal P (Rib-P)); HAV: hepatitis A virus; HBV: hepatitis B virus; HCV: hepatitis C virus; HEV: hepatitis E virus; Hb: hemoglobin; HIV: human immunodeficiency virus; HTLV: human T-lymphotropic virus; Ig: immunoglobulin; liver-specific autoantibodies: anti-AMA-M2, anti-M2-3E, anti-Sp 100, anti-PML, anti-gp 210, anti-LKM-1, anti-LC-1, anti-SLA/LP, anti-Actina F, anti-Ro-52; MB: molecular biology; respiratory viruses: influenza A and B, respiratory syncytial virus A/B, H1N1, H3N2, parainfluenza viruses 1-4, adenovirus, human metapneumovirus, human bocavirus, rhinovirus, enterovirus, coronaviruses (229E, HKU1, NL63, OC43), and SARS-CoV-2; SMAs: anti-smooth muscle antibodies; TSH: thyroid-stimulation hormone; Zoonosis screening: *Anaplasma phagocytophilum; Ehrlichia chaffeensis* and *Ehrlichia muris*; *Borrelia burgdorferi *sensu lato, *Borrelia miyamotoi, Borrelia hermsii; Coxiella burnetii; Babesia microti *and *Babesia divergens; Rickettsia *species; tick-borne encephalitis virus (TBEV)

Laboratory parameter	Results	Reference values
Respiratory viruses (MB)	Negative	-
Blood cultures	Negative	-
Zoonosis screening (blood)	Negative	-
Anti-Brucella abortus IgM and IgG	Negative	-
Leptospirosis (DNA blood and MB urine)	Negative	-
ANA	<1:160	<1:160
Anti-CCP	<0.4 U/mL	<0.4 U/mL
Rheumatoid factor	<10.0 UI/mL	<10.0 UI/mL
AMAs	<1:80	<1:80
SMAs	<1:40	<1:40
Liver-specific autoantibodies	Negative	-
ENAS	Negative	-
ANCA	<1:20	<1:20
HIV-1/2	Non-reactive	-
HAV	Past infection	-
HBV	Past infection	-
HCV	Non-reactive	-
HEV	Non-reactive	-
CMV	Past infection	-
Toxoplasma gondii	Past infection	-
EBV	Past infection	-
HTLV	Negative	-
Coxsackie IgM and IgG	Negative	-
Parvovírus IgM and IgG	Past infection	-
Echinococcus species	Negative	-
Fasciola hepatica	Negative	-
Schistosoma species	Negative	-
Taenia solium	Negative	-
Toxocara species	Negative	-
Trichinella spiralis	Negative	-
Strongyloides stercoralis	Negative	-
Entamoeba histolytica	Negative	-
TSH	3.35 mIU/L	0.27-4.20 mIU/L
C3	155 mg/dL	90-180 mg/dL
C4	32 mg/dL	12-36 mg/dL
IgA	284 mg/dL	78-312 mg/dL
IgG	786 mg/dL	650-1500 mg/dL
IgM	56 mg/dL	55-300 mg/dL

**Figure 1:**
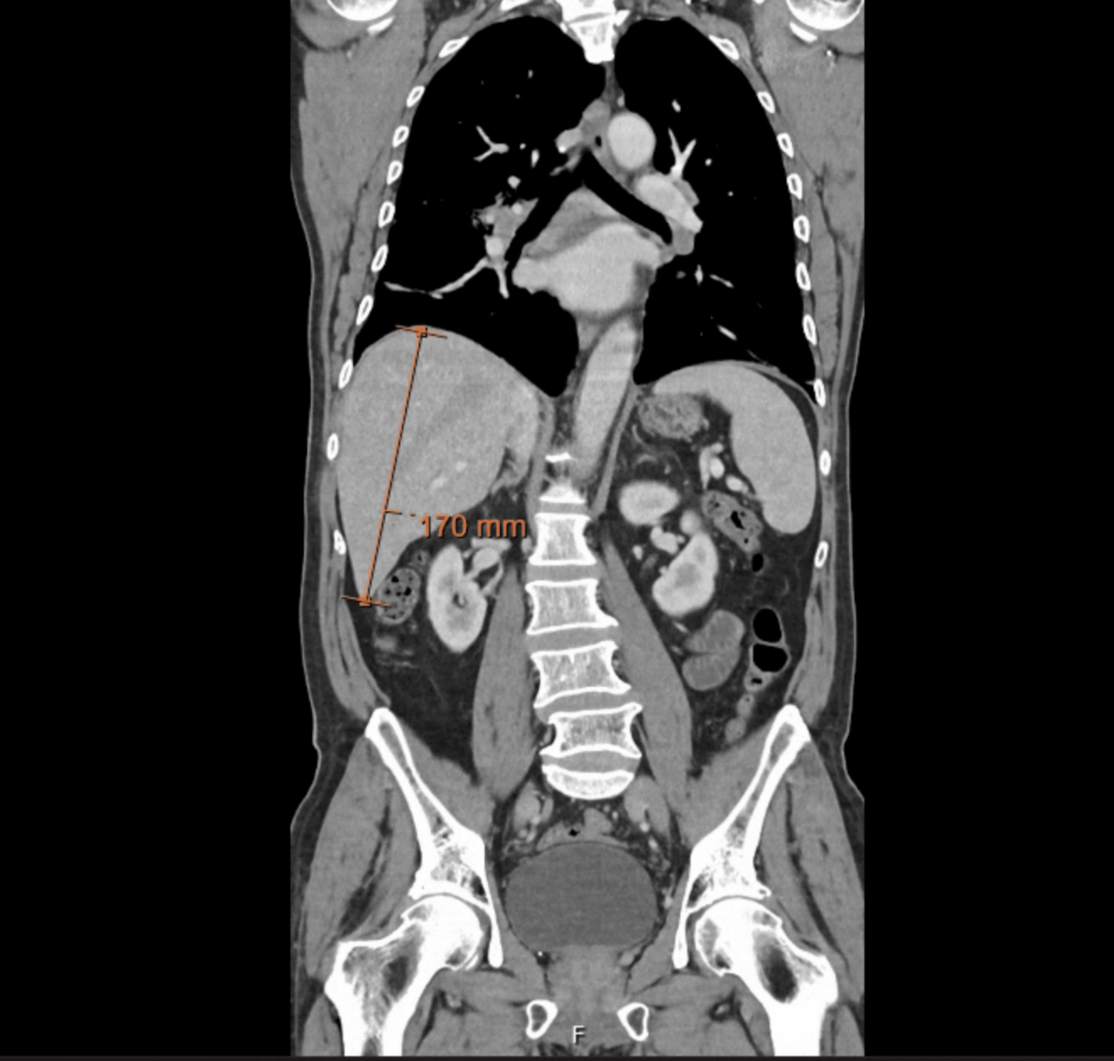
Coronal abdominopelvic computed tomography (CT) demonstrating hepatomegaly, with the right hepatic lobe measuring 17 cm (arrow).

**Figure 2:**
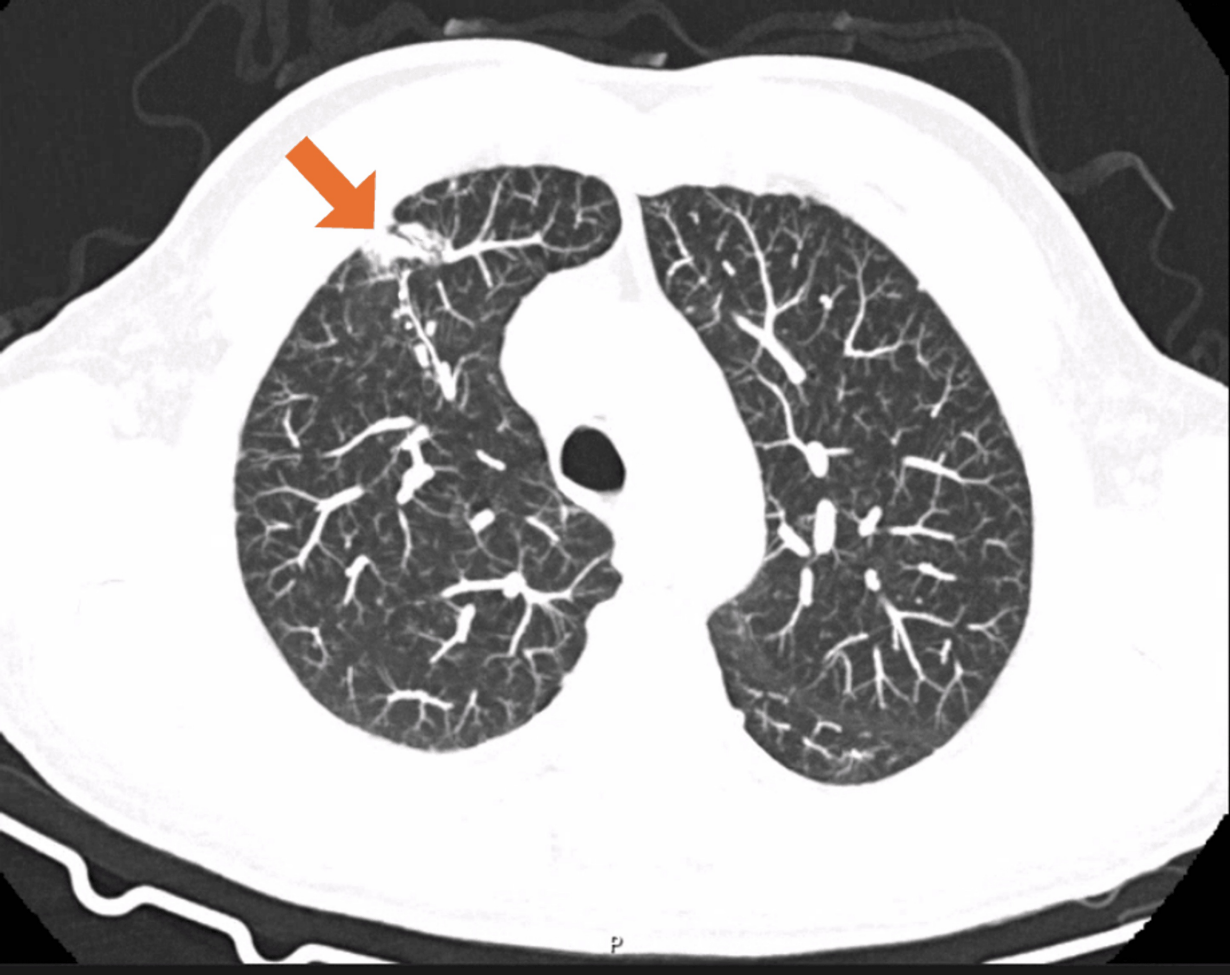
Axial chest computed tomography (CT) demonstrating subtle fibrotic changes in the upper lobes (arrow).

Abdominopelvic computed tomography (CT) showed hepatomegaly (right hepatic lobe measuring 17 cm), without other abnormalities (Figure 1). Chest CT revealed subtle upper-lobe fibrosis and mediastinal-hilar lymphadenopathy up to 16 mm (Figure 2). Abdominal ultrasound and magnetic resonance (MR) cholangiopancreatography were unremarkable. Transthoracic echocardiogram showed no evidence of valvular vegetations.

Throughout the diagnostic workup, the patient persisted with fever and inflammatory arthralgias. He developed normocytic normochromic anemia with elevated ferritin, consistent with a systemic inflammatory response. Liver enzymes and CRP remained elevated (Table 1). Blood cultures and zoonosis screening (including brucellosis and leptospirosis) were negative (Table 2).

**Figure 3:**
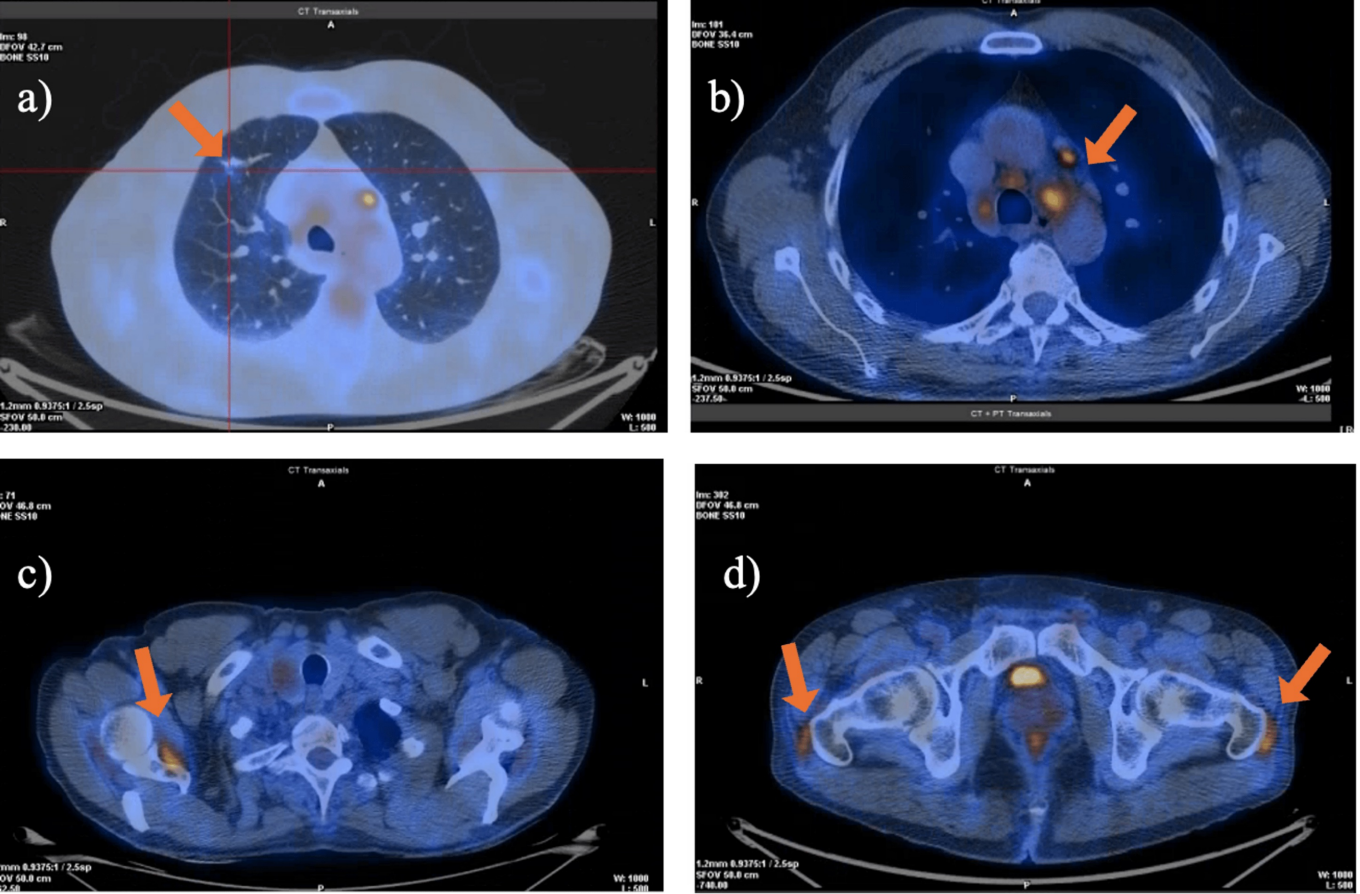
Whole-body 18F-fluorodeoxyglucose positron emission tomography-computed tomography (18F-FDG PET-CT). (a) Moderate FDG uptake (qSUVmax = 1.93) in a small right upper-lobe pulmonary density (arrow). (b) Hypermetabolic mediastinal and hilar lymph nodes involving the bilateral paratracheal, para-aortic, subaortic, subcarinal, and hilar stations (qSUVmax = 5.01 on the right) (arrow). (c) Symmetric FDG uptake in the bilateral glenohumeral and sternoclavicular joints and along the inferior scapular margins (arrow). (d) Increased FDG uptake over the bilateral femoral greater trochanters (arrows). qSUVmax: quantitative maximum standardized uptake value

Whole-body 18F-FDG PET-CT revealed moderate FDG uptake (quantitative maximum standardized uptake value (qSUVmax) = 1.93) in a small density in the right upper lung, along with hypermetabolic mediastinal and hilar lymph nodes involving bilateral paratracheal, para-aortic, subaortic, subcarinal, and hilar stations (qSUVmax = 5.01 on the right), showing a globally symmetric pattern. And, uptake in bilateral glenohumeral joints, sternoclavicular joints, inferior scapular margins, and bilateral femoral greater trochanters was compatible with granulomatous inflammatory disease (Figures 3a-3d). The symmetric distribution of hypermetabolic lymphadenopathy further strengthened suspicion for sarcoidosis and aided in excluding malignant etiologies.

Liver biopsy was negative for granulomas and malignancy. Bronchoscopy revealed no macroscopic abnormalities. Bronchial aspirate was negative for bacterial, mycobacterial, and fungal pathogens, including polymerase chain reaction (PCR) for *Mycobacterium tuberculosis*. Endobronchial and mediastinal lymph node biopsies (EBUS) were inconclusive. These nondiagnostic biopsies represented a known limitation in extrapulmonary sarcoidosis, where granulomas may be patchy or absent despite active disease. BAL showed lymphocytic inflammation with a CD4/CD8 ratio of 7.3, compatible with sarcoidosis.

After multidisciplinary discussion and exclusion of infectious, autoimmune, and neoplastic etiologies, the clinical features and BAL supported a diagnosis of sarcoidosis with hepatic and joint involvement. Prednisolone 20 mg/day was initiated, resulting in complete resolution of fever and arthralgias within one week and progressive normalization of liver enzymes, CRP, erythrocyte sedimentation rate (ESR), and ACE levels (Table 1).

The patient was discharged with clear clinical improvement, with scheduled outpatient follow-up.

## Discussion

Fever of unknown origin and inflammatory arthralgias are symptoms that, in isolation, seldom suggest sarcoidosis at first presentation. However, they are recognized manifestations of the disease, particularly when systemic involvement is present [1]. This case illustrates the diagnostic complexity of sarcoidosis, especially when initial symptoms are nonspecific, and biopsies fail to demonstrate granulomas. The coexistence of prolonged fever, inflammatory arthralgias, and a cholestatic pattern of liver enzyme abnormalities required a comprehensive diagnostic approach.

Hepatic sarcoidosis is often subclinical but may present with cholestatic liver enzyme elevation, while jaundice is uncommon. Bilirubin, albumin, and international normalized ratio (INR) typically remain normal, indicating preserved liver hepatic function despite significant inflammatory activity. Historical findings can vary widely, and granulomas can be patchy or absent, particularly in early or predominant extrapulmonary disease. As highlighted in recent literature, normal or nondiagnostic liver biopsies do not exclude hepatic sarcoidosis, particularly when clinical, biochemical, and radiologic features strongly align with the diagnosis [5,6].

Joint involvement occurs in up to 40% of cases and may precede pulmonary manifestations [7]. An elevated CD4/CD8 ratio in BAL is a sensitive marker of granulomatous inflammation, and values above 3.5 strongly support the diagnosis of sarcoidosis [8]. FDG PET-CT demonstrating symmetric FDG uptake in mediastinal-hilar lymph nodes further supported a granulomatous inflammatory process and helped exclude malignancy [9].

Although histological confirmation remains the diagnostic gold standard, current American Thoracic Society/European Respiratory Society/World Association of Sarcoidosis and Other Granulomatous Disorders (ATS/ERS/WASOG) guidelines acknowledge that sarcoidosis may be diagnosed without granulomas when clinical, analytical, and imaging features strongly support the disease and alternative causes have been rigorously excluded [2]. In this context, the integration of clinical, analytical, and imaging findings - combined with the rapid therapeutic response to corticosteroids - strongly supported the diagnosis and highlighted not only the inflammatory nature of the disease but also its potential reversibility when treated early.

The choice of a 20 mg/day prednisolone regimen aligns with ATS/ERS/WASOG recommendations, which support the use of lower-dose corticosteroids (20-40 mg/day) in cases of extrapulmonary sarcoidosis without significant pulmonary involvement or organ-threatening disease [10]. Given this patient’s minimal pulmonary findings and predominantly hepatic and musculoskeletal manifestations, the lowest effective dose within the recommended therapeutic range was selected. Although higher doses are traditionally used in pulmonary sarcoidosis, moderate dosing is considered appropriate in presentations such as this one.

Similar diagnostic challenges have been reported in cases where systemic manifestations predominate and histology is non-diagnostic [11,12]. This underscores the importance of maintaining a high index of suspicion for sarcoidosis and of adopting a multidisciplinary approach that values the coherence of global clinical, analytical, and imaging data, even when histology is non-contributory.

Long-term outpatient follow-up was not yet available at the time of manuscript preparation, which limits assessment of treatment duration, steroid tapering strategy, and radiologic evolution. Nevertheless, the complete clinical and biochemical response observed during hospitalization supports the adequacy of the chosen initial regimen.

## Conclusions

Sarcoidosis may present with nonspecific systemic symptoms such as fever and arthralgias, which can obscure the diagnosis in the absence of clear pulmonary involvement. When infectious, autoimmune, and neoplastic causes are excluded, the integration of clinical, laboratory, and imaging findings becomes crucial. This case highlights the importance of considering sarcoidosis in patients with persistent inflammatory symptoms of unclear origin and demonstrates how a multidisciplinary approach can support diagnosis even when histology is inconclusive.
